# Picocyanobacteria in the Chesapeake Bay: isolation, diversity, and adaptation

**DOI:** 10.1007/s42995-024-00271-9

**Published:** 2025-01-07

**Authors:** Feng Chen

**Affiliations:** Institute of Marine and Environmental Technology, University of Maryland Center for Environmental Science, Baltimore, MD 21202 USA

**Keywords:** Picocyanobacteria, Chesapeake Bay, *Synechococcus*, Subcluster 5.2, Estuary

## Abstract

Tiny unicellular cyanobacteria or picocyanobacteria (0.5–3 µm) are important due to their ecological significance. Chesapeake Bay is a temperate estuary that contains abundant and diverse picocyanobacteria. Studies of Chesapeake Bay picocyanobacteria in the past 20 years led to the finding of new members of subcluster 5.2 *Synechococcus*. They laid the foundation for revealing the ecophysiology, biogeography, genomics, and molecular evolution of picocyanobacterial in the Chesapeake Bay and other coastal estuaries. The Bay picocyanobacteria are known to better tolerate the changes in temperature, salinity, and heavy metals compared to their coastal and open-ocean counterparts. Many picocyanobacteria isolated from the Bay contain rich toxin–antitoxin (TA) genes, suggesting that the TA system may provide them with a genetic advance to cope with variable estuarine environments. Distinct winter and summer picocyanobacteria are present in the Bay, suggesting a dynamic seasonal shift of the picocyanobacterial community in the temperate estuary. While the Bay contains subcluster 5.2 *Synechococcus*, it also contains freshwater *Synechococcus*, *Cyanobium,* and marine *Synechococcus* due to river influx and the ocean’s tidal influence. Some Chesapeake Bay picocyanobacterial clades were found in the Bering Sea and Chukchi Sea, showing a link between the Bay and polar picocyanobacteria. Genomic sequences of estuarine picocyanobacteria provide new insight into the taxonomy and evolution of freshwater, estuarine, and marine unicellular cyanobacteria. Estuaries connect freshwater and marine ecosystems. This overview attempts to extend what we learned from Chesapeake Bay picocyanobacteria to picocyanobacteria in freshwater and marine waters.

## Freshwater and marine picocyanobacteria

Oxygenic photosynthesis on Earth started with cyanobacteria about 2.5 billion years ago (Karlusich et al. 2020; Schopf 2000). As ancient oxygenic photosynthetic prokaryotes, cyanobacteria have been used as model organisms to study photosynthesis, nitrogen and phosphorus metabolism, and molecular evolution. Unicellular cyanobacteria in size range 0.2–2 µm are a group of important picoplankton that contribute significantly to carbon fixation and biogeochemical cycling in the marine environment (Johnson and Sieburth 1979; Waterbury et al. 1979). A specific term, picocyanobacteria, was later used to describe this small unicellular cyanobacteria group (Fuller et al. 1998; Urbach et al. 1998). Picocyanobacteria have been isolated from diverse habitats, including lakes, hot springs, rice paddies, low salinity brine ponds, marine sediments, and seawater since the 1950s (Rippka et al. 1979). The ability to grow cyanobacteria in solid culture media allowed early researchers to obtain clonal cyanobacterial isolates from different aquatic environments (Stanier et al. 1971). Three major genera, *Cyanobium*, *Synechococcus,* and *Prochlorococcus* dominate the culture collection of aquatic picocyanobacteria. *Cyanobium* is commonly present in freshwater and brackish water, *Synechococcus* is widely distributed from marine to freshwater ecosystems, and *Prochlorococcus* is restricted to the warm oligotrophic ocean.

The ecological significance of picocyanobacteria was not fully recognized in the late 1970s when high cell densities of planktonic cyanobacteria (presumably *Synechococcus*) were found in the open ocean by Waterbury et al. (1979). A few years later, another group of Chroococcoid cyanobacteria (prochlorophytes) was found to be predominant in the open ocean (Chisholm et al. 1988). The finding that picocyanobacteria (mainly *Synechococcus* and *Prochlorococcus*) contribute significantly to phytoplankton biomass and primary production in the ocean catalyzed later studies of picocyanobacteria in the marine environment (Flombaum et al. 2013; Goericke and Welschmeyer 1993; Li and Wood 1988; Li 1994; Liu et al. 1997; Olson et al. 1990; Partensky et al. 1999; Platt et al. 1983; Scanlan et al. 2009; Scanlan 2012; Waterbury et al. 1986; Zwirglmaier et al. 2008). Marine *Synechococcus* strains from coastal and open-ocean water have been isolated and characterized (Waterbury et al. 1986; Waterbury and Willey 1988; Ahlgren and Rocap 2006; Toledo and Palenik 1997; Toledo et al. 1999). The genetic diversity of *Prochlorococcus* and *Synechococcus* and their niche partitioning in the ocean were explored based on the *rpoC1* gene (Ferris and Palenik 1998). Marine *Synechococcus* was originally divided into three major groups, marine clusters A, B, and C (Waterbury et al. 1986; Waterbury and Rippka 1989). They were reclassified as subcluster 5.1 and 5.2 based on their morphological, physiological, genetic properties/characters, and phylogenetic analysis (Herdman et al. 2001). Subcluster 5.3 was not defined by Herdman et al. in 2001, likely due to the lack of 16S rRNA gene sequences, it was then defined later based on phylogenomics (Dufresne et al. 2003). Subcluster 5.1 is equivalent to marine cluster A. While subcluster 5.2 is claimed to be equivalent to marine cluster B and contains three coastal strains, WH5701, WH8007, and WH8101, with WH5701 as a reference strain, it has not been proved based on the 16S rRNA gene phylogeny (Herdman et al. 2001). In the phylogenetic tree, subcluster 5.2 only included one reference sequence from WH8010 (Herdman et al. 2001); thereof, it is an ill-defined phylogenetic group. The grouping of strains WH5701, WH8007, and WH8101 into subcluster 5.2 without phylogenetic support (Herdman et al. 2001) generated confusion for later studies. Moreover, subcluster 5.2 was initially considered to contain *Synechococcus* species which are non-motile and lack phycoerythrin (Herdman et al. 2001). Later studies showed that subcluster 5.2 also includes *Synechococcus* isolates with phycoerythrin and mobility (Chen et al. 2004, 2006). In the early days (early 2000’s), subclusters 5.1, 5.2, and 5.3 mainly included *Synechococcus* isolated from marine environments.

In freshwater systems, *Synechococcus* and *Cyanobium* are among the most dominant picocyanobacteria (Callieri et al. 2013). Many picocyanobacterial strains have been isolated from different lakes, such as Lake Biwa in Japan (Maeda et al. 1992), Lake Balaton in Hungary, Lake Maggiore in Italy (Callieri et al. 1996), Lake Constance (Ernst et al. 1995), deep subalpine lakes located in Upper Austria (Crosbie et al. 2003), and four Mazurian lakes in Poland (Jasser et al. 2011). These freshwater picocyanobacterial isolates belong to several major genetic lineages such as subalpine I and II, Lake Biwa (or Group E), *Cyanobium gracile* cluster, Group A, B, Cz, H, I and M based on the 16S RNA gene phylogeny (Ernst et al. 2003). Many were grouped under the *Cyanobium* cluster (Ernst et al. 2003). *Synechococcus* can also be present in the freshwater ecosystem. It was recognized 20 years ago, *Synechococcus* should be reclassified into several independent taxonomic units (Honda et al. 1999) and has undergone niche-adaptive evolution (Urbach et al. 1998). The taxonomy of picocyanobacteria has been constantly challenged with newly discovered cultures and environmental sequences in the past 20 years. Extensive work has been done to study the chromatic adaptation and evolution of phycobiliprotein in non-marine environments (Crosbie et al. 2003; Haverkamp et al. 2008; Everroad and Wood 2012; Sánchez-Baracaldo et al. 2019).

The genetic diversity of freshwater and marine picocyanobacteria has been investigated using various gene makers, i.e., the 16S rRNA gene, ITS, RNA polymerase gene, etc. New genotypes and phyletic clades emerge when diverse aquatic habitats are investigated, reflecting the broad niche adaptation of picocyanobacteria. Numerous articles have provided a comprehensive review or introduction to the genetic diversity and evolution of freshwater and marine picocyanobacteria at both the gene and genomic levels (Scanlan and West 2002; Rocap et al. 2002; Ivanikova et al. 2007; Dufrense et al. 2008; Sánchez-Baracaldo et al. 2008; Scanlan et al. 2009; Ahlgren and Rocap 2012; Everroad and Wood 2012; Huang et al. 2012; Callieri et al. 2013; Coutinho et al. 2016a, b; Callieri 2017; Di Cesare et al. 2018; Sánchez-Baracaldo et al. 2019; Salazar et al. 2020, Cabello-Yeves et al. 2022). It has been realized that picocyanobacteria in the estuarine environment are important and currently there is no review on estuarine picocyanobacteria.

## Chesapeake Bay estuary

**Fig. 1:**
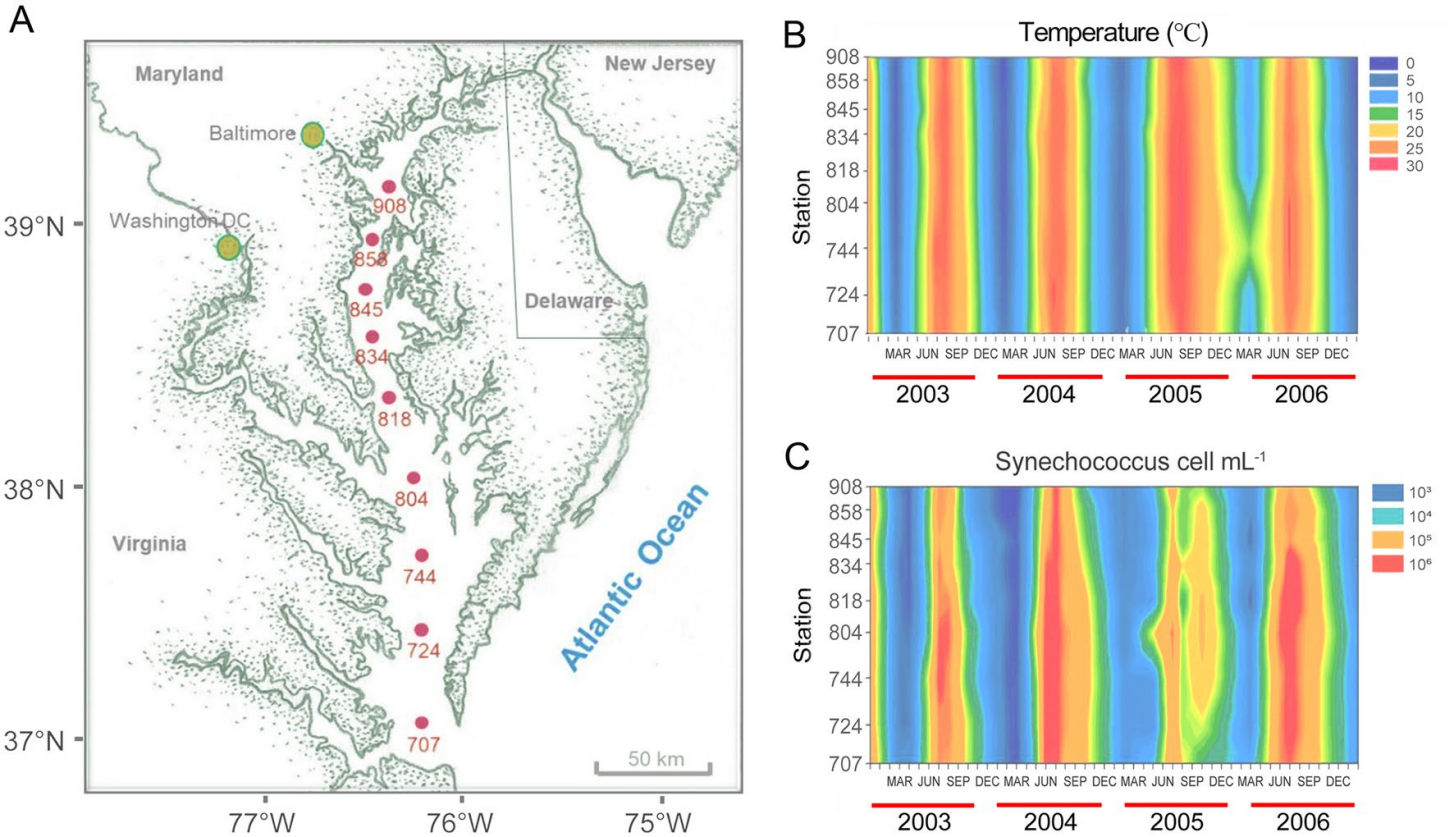
Nine sampling sites of the Chesapeake Bay for the Microbial Observatories Viral Ecology (MOVE) project research cruises between 2003 and 2006 (**A**), the contour plot of surface water temperature (**B**) and picocyanobacterial cell counts (**C**) were measured during the MOVE project

The Chesapeake Bay is the largest estuary in the United States (Fig. 1A), with 16 million people living and impacting the watershed (Harding et al. 2016). The Bay is approximately 320 km long with an average depth of 6.4 m. Mixing of freshwater and the Atlantic seawater creates a strong salinity gradient from the northern Bay to the southern Bay. Chesapeake Bay is a productive estuary that supports the growth of different aquatic species including economically important species like seabass, crabs, oysters, etc. (www.chesapeakebay.net). A long (~ 180 days) water residence time has been reported for the Chesapeake Bay (Du and Shen 2016). The modern Chesapeake Bay was formed by the most recent rise in sea level and is less than 10,000 years old (Schubel and Pritchard 1986). The Susquehanna River contributes nearly half of fresh water and impacts the bay ecology significantly (Harding et al. 2016). The increase of nutrient loading (nitrogen and phosphorus) after World War II in the Bay (Boynton et al. 1995) often stimulated the phytoplankton blooms which resulted in hypoxia in summer (Sharp et al. 2009).

## Picocyanobacteria in the Chesapeake Bay

Phytoplankton studies in the Bay began about a century ago (Wolfe et al. 1926), and most of the earlier studies focused on large phytoplankton, which can be identified under a light microscope. Later studies showed that small phytoplankton, nanophytoplankton, and picophytoplankton are responsible for a large portion of total phytoplankton biomass and productivity (McCarthy et al. 1974; Malone et al. 1991). Picocyanobacteria are the predominant portion of picophytoplankton. Most early studies of picocyanobacterial focused on the abundance, growth, productivity, and spatial and temporal distribution of picocyanobacteria. Chesapeake Bay is a temperate estuary with strong seasonal changes in temperature (Fig. 1B). Picocyanobacteria were found to be most abundant in summer and least abundant in winter (Affronti 1990; Ray et al. 1989). This seasonal pattern is mirrored by picoplankton primary production being highest in July at 55.6% and lowest at 2.3% in January (Affronti and Marshall 1994). During a 4-year survey (2003–2006) for the Microbial Observatories Viral Ecology (MOVE) project, we found that picocyanobacterial abundance exhibited a similar inter-annual pattern in the Bay (Fig. 1C). Cell counts of picocyanobacteria exceeded 1 million cells per mL during the summer ‘bloom’ season and dropped below 1000 cells per ml in winter (Wang et al. 2011).

The ratio of phycobiliproteins, phycocyanin (PC)-rich and phycoerythrin (PE)-rich picocyanobacterial strains has been used to investigate the distribution of different pigment types of Chesapeake Bay picocyanobacteria over time and space. PC-rich picocyanobacteria were eight times more prevalent than the PE-rich picocyanobacteria based on epifluorescence microscopy counting (Ray et al. 1989), a striking contrast to the open ocean where PE-rich picocyanobacteria dominate (Campbell and Carpenter 1987). Furthermore, PC-rich picocyanobacteria dominated surface waters in August 1988, accounting for 73.8% of surface picophytoplankton (Affronti and Marshall 1993). In the winter (January 1990), benthic PE-rich picocyanobacteria were more productive and comprised 65.4% of benthic picophytoplankton (Affronti and Marshall 1993). These early studies of Chesapeake Bay picocyanobacteria mainly focused on the spatiotemporal distribution of their abundance, pigmentation, biomass, and productivity. Little was known about the ecophysiology and genetic diversity of picocyanobacteria in the Bay back then. We need to find out what types of picocyanobacteria live in the Bay. With these questions in mind, we began to isolate picocyanobacteria from the Chesapeake Bay in the early 2000s.

## The Chesapeake Bay harbors unique clades of *Synechococcus* belonging to subcluster 5.2

**Fig. 2:**
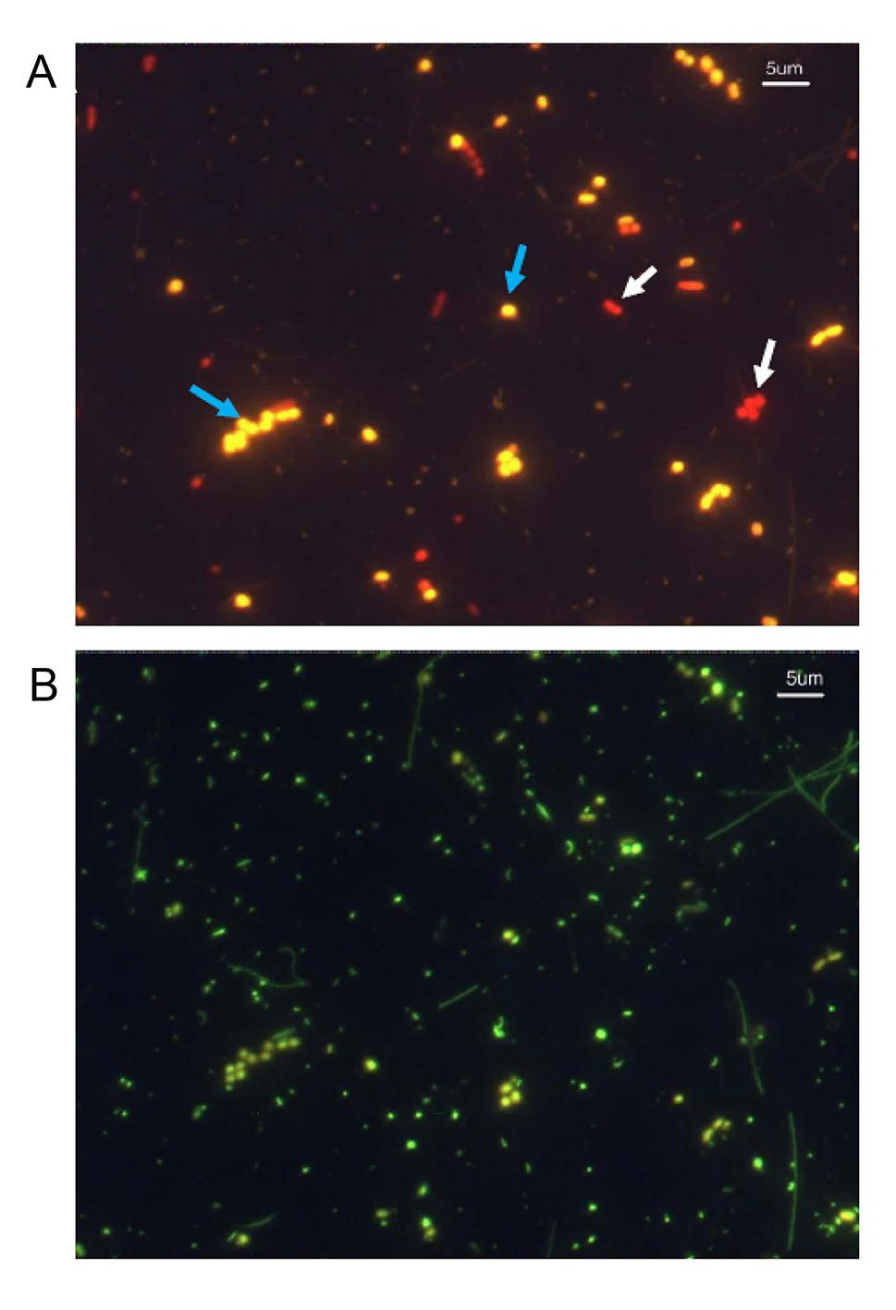
Epifluorescent micrographs of picocyanobacteria (upper panel) and the total microbial community (lower panel) under the same view field with different excitation wavelengths (green vs blue excitation). Picocyanobacterial PC-rich and PE-rich cells are shown with white and blue arrows, respectively. The water sample was collected from the Chesapeake Bay in the summer

**Fig. 3:**
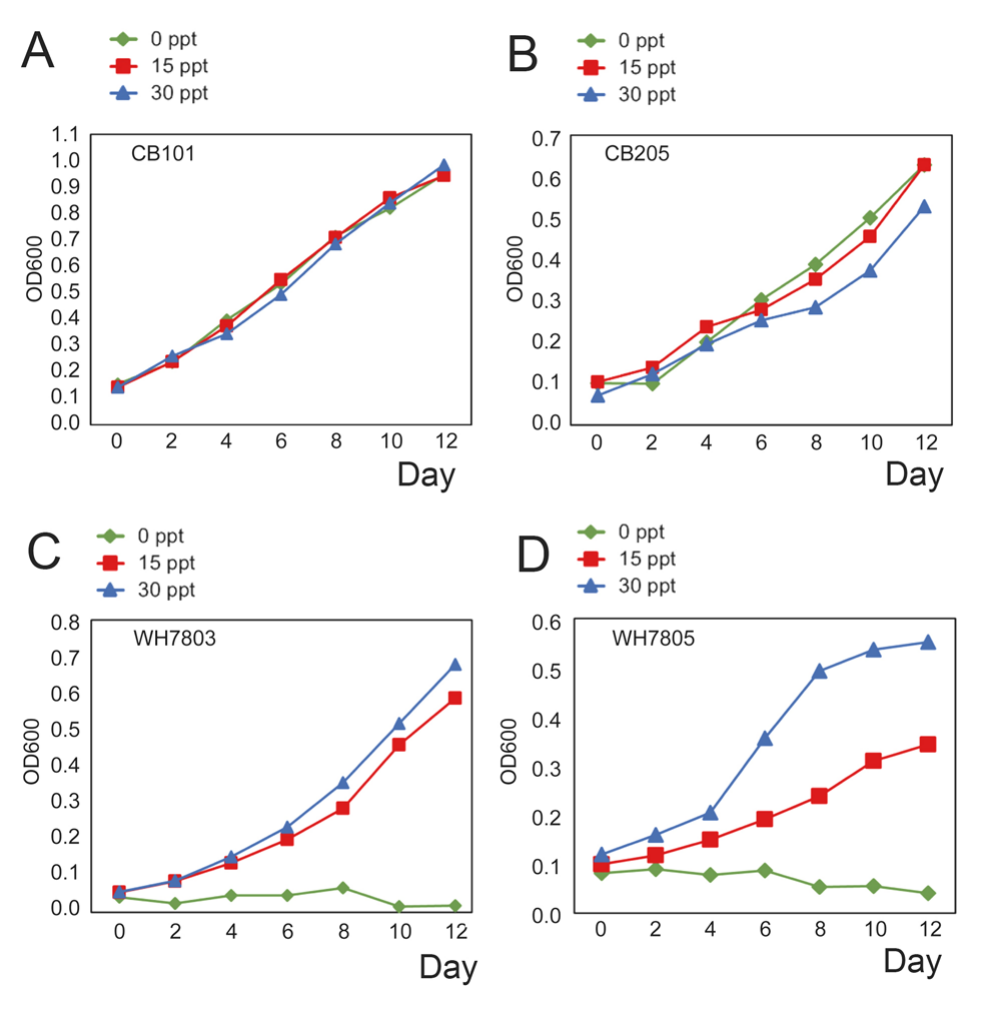
Growth comparison of four *Synechococcus* strains at three different salinity levels (0, 15, and 30ppt, respectively): **A** Chesapeake Bay strain CB0101; **B** Chesapeake Bay strain CB0205; **C** coastal strain WH7803; and **D** open-ocean strain WH7805. Four other CB trains CB0104, CB0201, CB0208, and CB0210 have similar growth curves like CB0101 and CB0205 (data not shown)

Thirteen picocyanobacteria were isolated from various locations in the Chesapeake Bay, including the Baltimore Inner Harbor, middle, and lower Bay, mainly during the summer months (Chen et al. 2004). Because salinity varies significantly from the upper to lower Chesapeake Bay, we adjusted media salinity to simulate the salinity of water samples. Microscopic observation of these isolates showed that they were unicellular cyanobacteria with a coccoid or rod shape and cell sizes between 1 and 3 µm. Among these 13 isolates, 7 strains were PC-rich and 6 were PE-rich *Synechococcus*. Five motile strains were also identified in these *Synechococcus* cultures. During isolation, 80%–90% of colonies grown on the plates were PC rich for the samples collected from the Baltimore Inner Harbor and the upper Chesapeake Bay, while 56%–65% of colonies on the plates were PC rich for the samples collected from the lower bay. A higher number of PC-rich picocyanobacteria in the upper bay, and a lower number of PC-rich picocyanobacteria in the lower bay were also evident based on enumeration by epifluorescence microscopy (Chen et al. 2004). The upper bay has relatively low salinity due to river influx, while the lower bay has relatively high salinity as it connects to the Atlantic Ocean (Fig. 1A). Salinity is usually in the range of 5–10 (Practical Salinity Unit) in the Baltimore Inner Harbor or the upper bay, 10–20 in the middle bay, and 20–30 in the lower bay. In general, picocyanobacteria can make up approximately 20%–30% of total bacterial counts (Fig. 2) and 20%–40% of total phytoplankton chlorophyll a in summer Chesapeake Bay (Wang et al. 2011). Chesapeake Bay *Synechococcus* isolates can grow in a culture medium with a wide salinity range (0–30). In contrast, many coastal and open-ocean *Synechococcus* do not grow in 0 salinity (Chen et al. 2004). A growth comparison between two Chesapeake Bay *Synechococcus* (CB0101 and CB0205) and two offshore *Synechococcus* strains (WH7803 and WH7805) is shown in Fig. 3.

The phylogenetic position of these *Synechococcus* strains was first illustrated based on the ribulose-1, 5-bisphosphate carboxylase-oxygenase (RuBisCO) large subunit gene (*rbcL*) sequences (Chen et al. 2004). The *rbcL* gene is conserved among photosynthetic organisms. The *rbcL* phylogeny showed that the majority of Chesapeake Bay *Synechococcus* formed its own clade next to the marine cluster A *Synechococcus*, and they contain Form IA RuBisCO, together with marine cluster A *Synechococcus* and marine *Prochlorococcus*. Freshwater *Synechococcus* strains carry Form IB RuBisCO. This result suggests that Chesapeake Bay *Synechococcus*, marine *Synechococcus,* and *Prochlorococcus* share the same origin of RuBisCO. We also found that *Synechococcus* strains WH5701 and WH8007 do not cluster together based on the *rbcL* gene phylogeny, suggesting that these two earlier isolates in marine cluster B or subcluster 5.2 (Herdman et al. 2001) are not closely related. WH5701 was isolated from Long Island Sound, while WH8007 was isolated from the Gulf of Mexico. WH8007 is more closely related to our Chesapeake Bay isolates than WH5701, suggesting that WH5701 may not be a member of subcluster 5.2. This observation is consistent with the phylogenetic analysis based on internal transcribed spacer (ITS) sequences (Chen et al. 2006, also see the following section). WH8007 grows well at salinities ranging from 18 to 30 (Lu et al. 2001); however, it appears that this strain has not been widely included in the phylogenetic studies of picocyanobacteria. Instead, WH5701 has been widely used as the reference strain for subcluster 5.2 (Callieri et al. 2013; Dufresne et al. 2003; Everroad and Wood 2012; Scanlan et al. 2009).

**Fig. 4:**
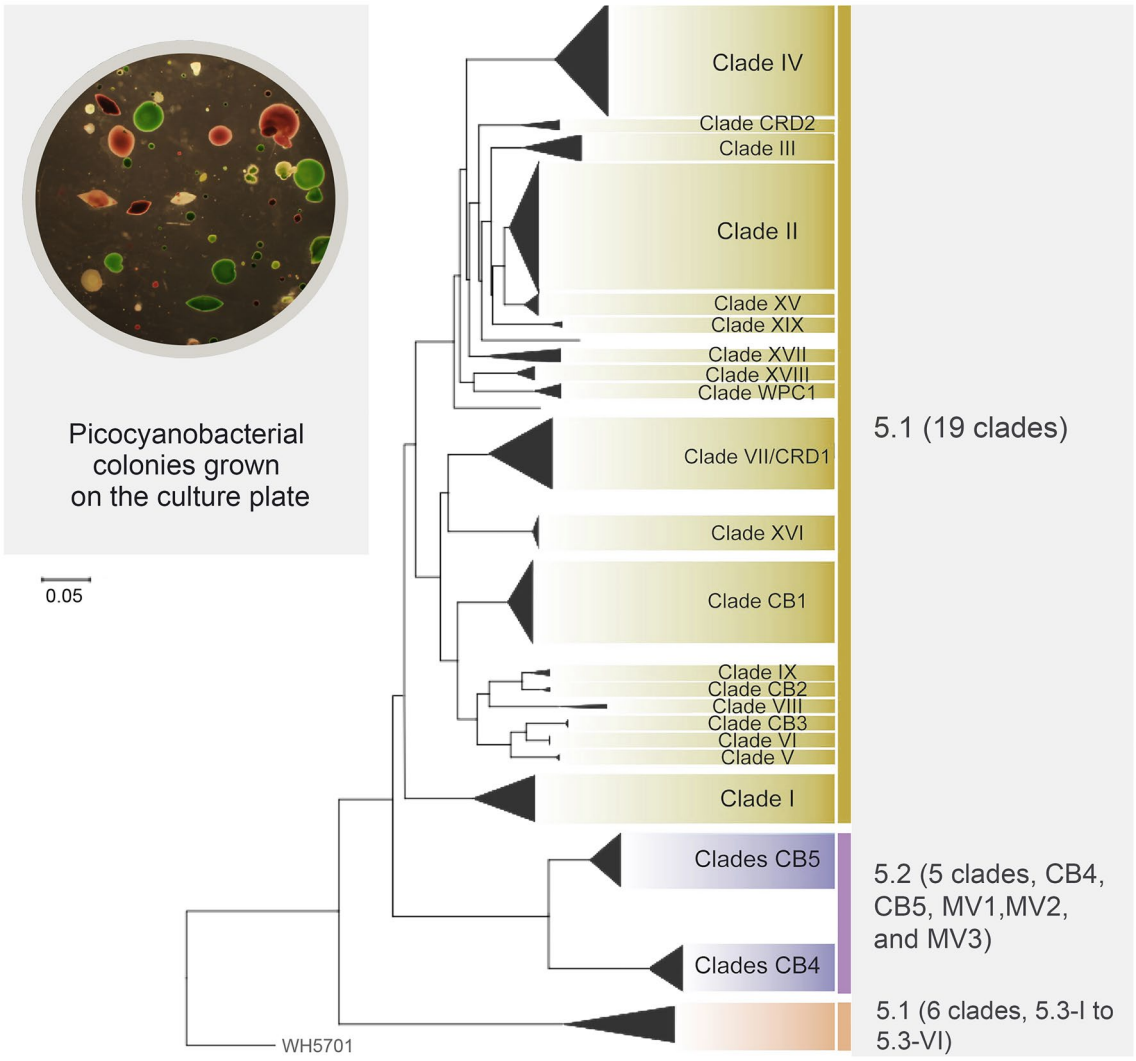
Schematic phylogeny of subcluster 5.1, 5.2, and 5.3 based on the ITS gene sequences. Clades MV1, MV2, and MV3 were reported by Hunter-Cevera et al. (2016)

Based on the ITS phylogeny, 11 Chesapeake Bay *Synechococcus* isolates fell into 2 clades, CB4 and CB5, which belong to subcluster 5.2, suggesting that *Synechococcus* living in the estuarine environment contain unique genotypes/ecotypes (Chen et al. 2006, Fig. 4). This study also reclassified subcluster 5.2. We proposed that WH8007 but not WH5701 should be a reference for subcluster 5.2 because WH5701 is closely related to Subalpine subcluster II and does not represent many estuarine *Synechococcus* (Chen et al. 2006). Clustering WH5701 into Subalpine subcluster II has been well-supported in later studies (Huang et al. 2014; Xu et al. 2015). WH8101 was removed from subcluster 5.2 and placed in subcluster 5.1 (Rocap et al. 2002). Subcluster 5.1 includes many isolates from the open ocean and is a well-supported phylogenetic group with at least 19 clades found (Fig. 4). Subcluster 5.3 *Synechococcus,* first defined by Dufrense et al. (2008), does not contain many culture isolates. A survey in different oceans identified 40 subcluster 5.3 clones, and many appear to be in deeper water (100–150 m) (Huang et al. 2012). At least six different clades (5.3-I to 5.3-VI) have been reported for subcluster 5.3 (Fig. 4). We were only able to detect cyanobacterial clones in the September clone libraries, but not in the March clone libraries likely due to the low cyanobacterial abundance in the cold season (Chen et al. 2006). In September, subcluster 5.2 *Synechococcus* prevailed above the middle bay, while subcluster 5.1 *Synechococcus* was prevalent below the middle bay (Chen et al. 2006).

## Presence of subcluster 5.2 *Synechococcus* in other coastal estuaries

**Fig. 5:**
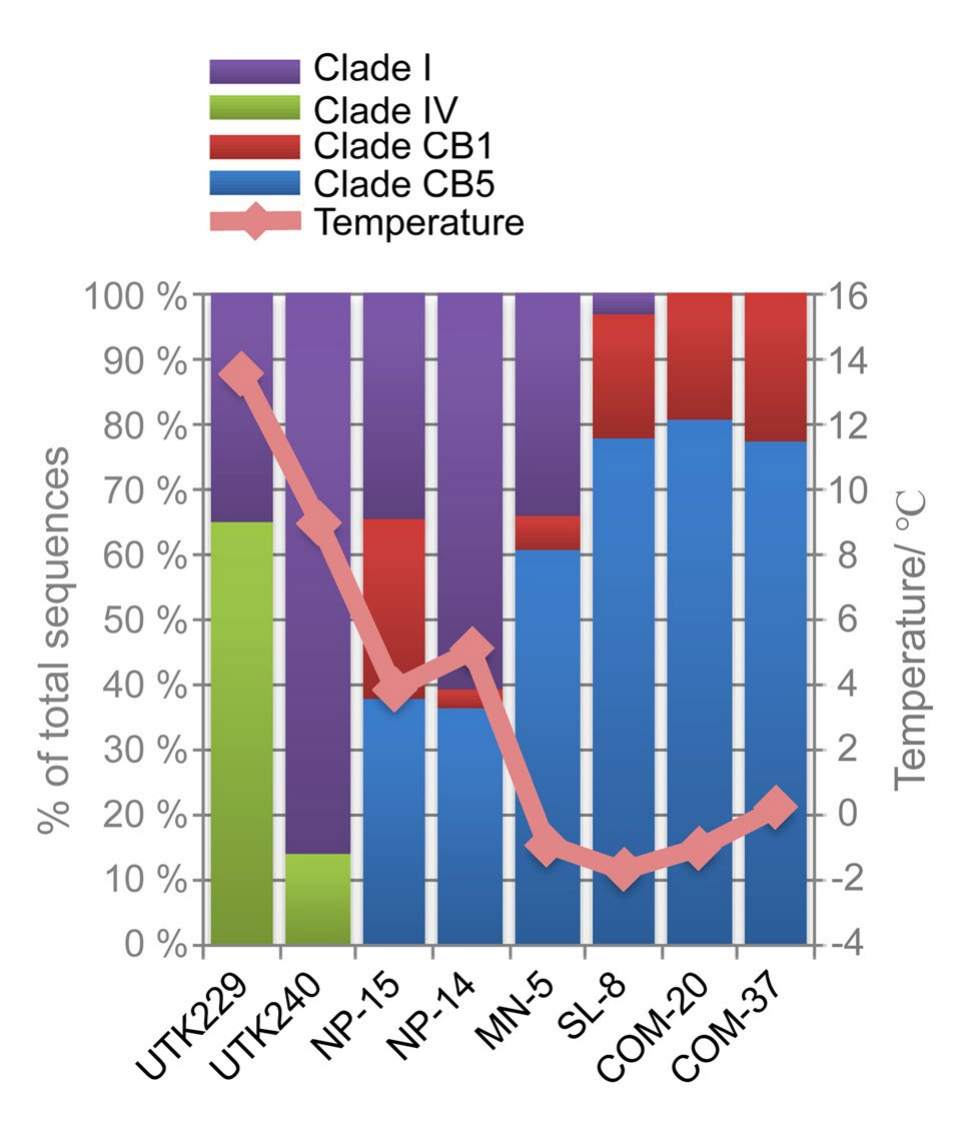
Relative sequence abundance of *Synechococcus* genotypes (clades I, IV, CB1, and CB5) in high latitude oceans. Sequences were detected by PCR amplification of the ITS region using primers specific for picocyanobacteria (Huang et al. 2012)

Later studies based on the community analysis of picocyanobacteria confirmed the presence of subcluster 5.2 *Synechococcus* in estuarine and near-shore waters. Environmental sequences of subcluster 5.2 were reported in the East China Sea (Choi and Noh 2009), estuarine water in the Sea of Okhotsk (Jing et al. 2009), the coastal estuary of Hong Kong (Xia et al. 2015), the Baltic Sea (Larsson et al. 2014), the Bering Sea and Chukchi Sea (Huang et al. 2012), the coastal estuaries near Massachusetts (Hunter-Cevera et al. 2016; Mackey et al. 2017). Clades CB4 and CB5 were found in the coastal water of Massachusetts, together with three other new clades (MV1, MV2, and MV3) of subcluster 5.2 (Hunter-Cevera et al. 2016). Clade CB5 of subcluster 5.2 dominated the estuarine waters of Sippewissett salt marsh (Massachusetts, US) in the late summer (Mackey et al. 2017). It is striking to see that subcluster 5.2 *Synechococcus* (CB5 clade) makes up the vast majority (*ca.* 80%) of picocyanobacteria in the Chukchi Sea during the summer when the water temperature is about 0 ºC (Huang et al. 2012). A clear shift of *Synechococcus* genotypes from Clade I and IV of subcluster 5.1 to Clade CB1 and CB5 from the Bering Sea to the Chukchi Sea was found (Fig. 5). Clades I and IV of subcluster 5.1 are known to co-occur in the high latitude ocean (Zwirglmaier et al. 2008). Clade V was not detected in the samples with water temperature lower than 5 ºC, but Clade I was present in the samples with water temperature between 5 and – 1 ºC (Fig. 5). This result suggests that *Synechococcus* in clade I of subcluster 5.1 can occupy colder water than clade V. CB1, a clade of subcluster 5.1 first identified in the Chesapeake Bay co-occurs with CB5 of subcluster 5.2 in the subzero waters of the Chukchi Sea. The Chukchi Sea is a shallow shelf sea (ca. 50 m deep) that can be influenced by freshwater from ice melting. The shift of picocyanobacteria in the high latitude and polar region is interesting, but we know little about how they survive the dark and cold winter in the polar water. The abundance of picocyanobacteria is often low (< 1000 cells per ml) in winter or a cold region like the polar ocean, and this makes it difficult to detect them using metagenomics or 16S rRNA gene clone library. With the universal bacterial 16S rRNA gene primers, all or most 16S rRNA gene sequences could be affiliated with chloroplast 16S rRNA gene from phytoplankton, but not with picocyanobacteria. The use of picocyanobacteria-specific PCR primers based on the ITS region avoided this problem and allowed us to investigate picocyanobacterial diversity in low-temperature environments like the polar ocean or during the winter season (Huang et al. 2012).

In a temperate ecosystem like the Chesapeake Bay, the water temperature can fluctuate between 1 and 28 ºC (Kan et al. 2006). Temperature appears to be the most critical factor influencing the seasonal patterns of microbial communities in the Bay (Kan et al. 2006, 2007; Wang et al. 2020). We also found that *Synechococcus* populations present in winter are distinct from those in the summer (Cai et al. 2010). Clades CB1, CB3, CB4, and CB5 were detected in the summer, and only clades CB6 and CB7 were detected in the winter. CB6 clones are closely related to CC9311 and WH8020 which are the members in clade I of subcluster 5.1. CB7 is a sister clade related to subalpine cluster I, *C. gracile* cluster, and Lake Biwa cluster, common picocyanobacteria found in the freshwater system.

## Winter Chesapeake Bay isolates—a link to picocyanobacteria in the Baltic Sea

**Fig. 6:**
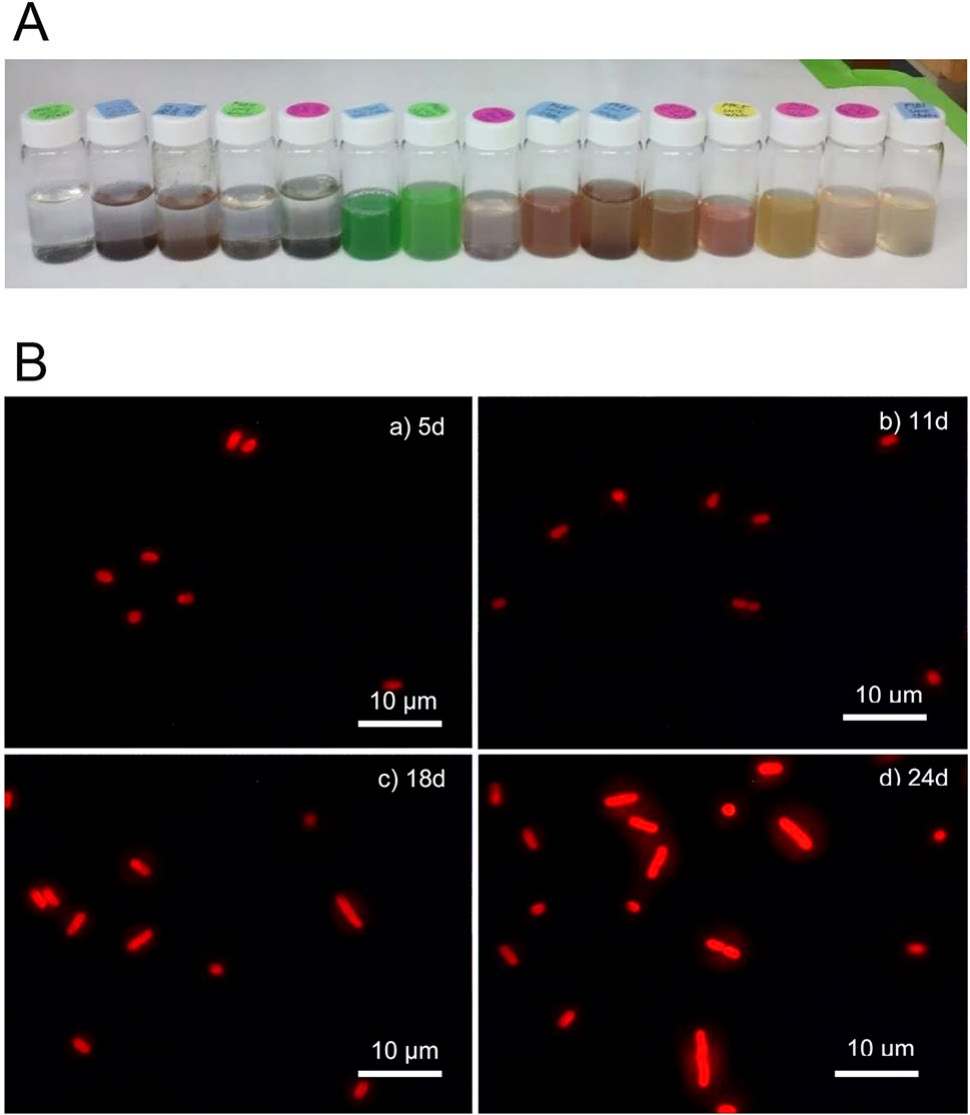
Clonal isolates of cyanobacteria from the Baltimore Inner Harbor during the winter season (**A**). Cultures were isolated using the SN medium with 15 salinity. Variable morphology of the Bornholm Sea cluster strain CBW1001. **B** Cell morphology of a winter isolate CBW1001 at different growth stages at 23 °C: **a** middle exponential growth phase; **b** late exponential growth phase; **c** stationary phase; and **d** extended stationary phase

The Bay *Synechococcus* strains obtained in early 2000 were isolated during the summer season, and most of them are subcluster 5.2 *Synechococcus*. While the summer isolates show their marine origin based on the *rbcL* gene phylogeny (Chen et al. 2004), the winter isolates show a strong connection with freshwater and brackish picocyanobacteria. Seventeen picocyanobacterial strains were isolated from the Baltimore Inner Harbor during the winter season (December 2010 and February 2011) (Xu et al. 2015). The water temperature varied between 2 and 8 °C, and the salinity fluctuated between 6 and 19 during this period. Seven PC-rich and ten PE-rich strains were recovered. The winter isolates are colorful and exhibit blue–green, yellowish, brown, and pink pigmentation, suggesting a wide chromatic adaptation of winter picocyanobacteria (Fig. 6A, also refer to Xu et al. 2015). They were named *Synechococcus* based on their small coccoid or short rod cell shape.

The winter picocyanobacteria can tolerate a wide temperature range. The effects of temperatures (4, 10, 15, 23, 25, or 28 °C) on the growth of winter and summer Chesapeake Bay *Synechococcus* strains, and their coastal and open-ocean counterparts were compared (Xu et al. 2015). Except for CBW1108, all the winter *Synechococcus* (8 representative strains) could grow slowly at 4 °C and 10 °C, but none of the coastal and open-ocean *Synechococcus* grew at these temperatures. The winter isolates maintained slow growth or prolonged dormancy at 4 °C and resumed normal growth at room temperature. This phenomenon was not observed in open-ocean *Synechococcus*. Interestingly, several *Synechococcus* strains in the Bornholm Sea cluster exhibited different cell lengths during the exponential growth. For example, the cell length of CBW1001 varies from 1.5 to 9 µm at different growth stages (Fig. 6B). Many winter Chesapeake Bay (CB) strains displayed a 2–3-fold cell enlargement during prolonged exposure to 4 °C. Cell enlargement or increase in cell volume (not elongation) under the cold condition has been reported in freshwater *Synechococcus* (Jezberová and Komárková 2007). Cell elongation (up to 50-fold) was found later in freshwater *Synechococcus* PCC 7942 during the stationary phase at room temperature, and the elongation of *Synechococcus* has been related to the stress induced by phosphorus limitation (Goclaw-Binder et al. 2012).

**Fig. 7:**
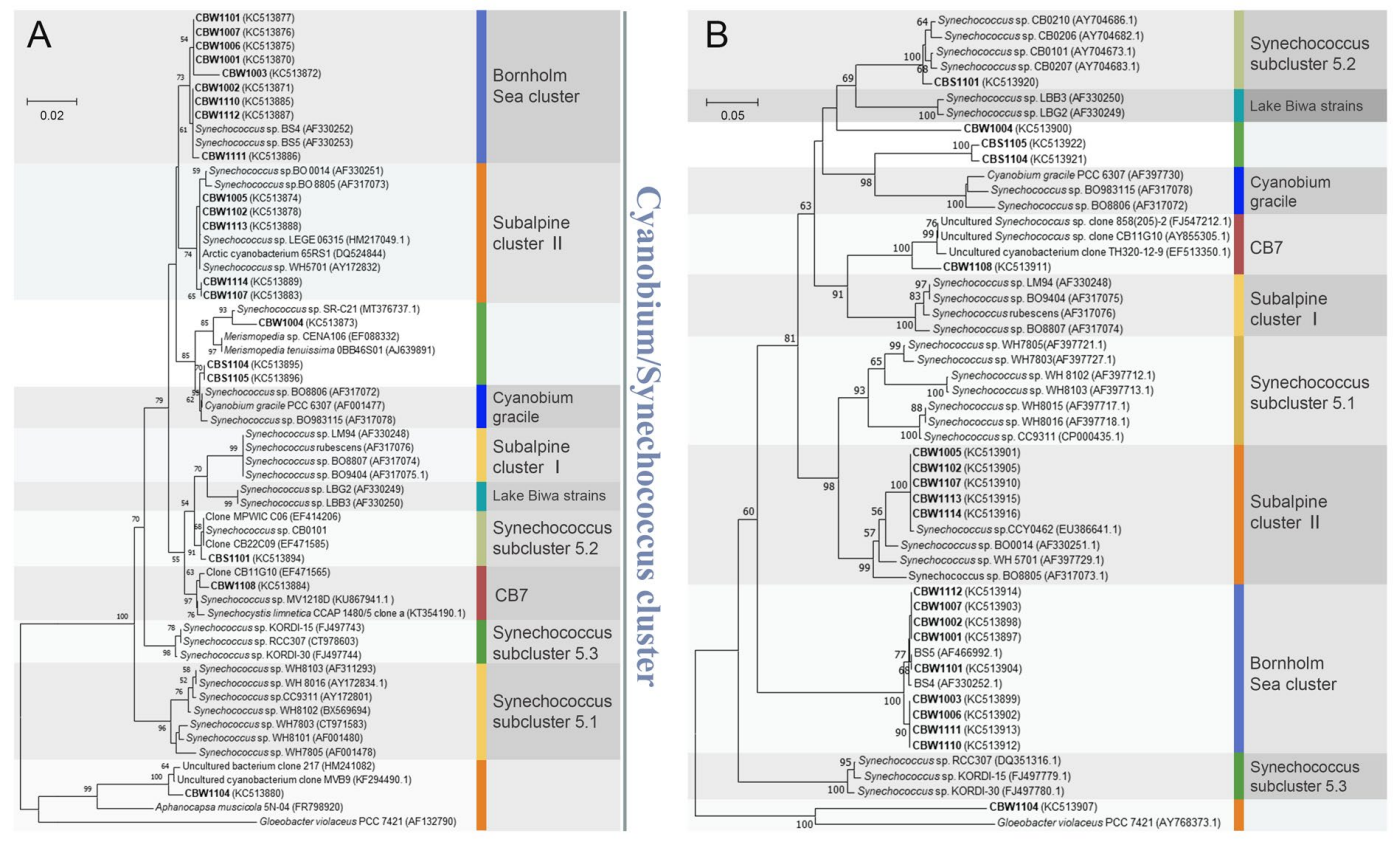
Neighbor-joining trees based on the 16S rRNA gene (**A**) and ITS (**B**) sequences, with bootstrap = 1000. Strains in bold were isolated from the Chesapeake Bay. CBW strands for Chesapeake Bay Winter. The partial 16S rRNA gene was PCR amplified using the primers CYA106F (50-CGGACGGGTGAGTAACGCGTGA-30) and CYA781R (50-GACTACWGGGGTATCTAA TCCCWTT-30; Nübel et al. 1997). The ITS region was amplified using the primers 16S-1345f (5ʹ-GCCACGCCCGAAGCCGTTACT-3ʹ) and 23S-2r (5ʹ-GAGCCCTTTGTAGCTTGACC-3ʹ). The Neighbor-Joining method assuming the Kimura-2-parameter model was used in the construction of the 16S tree, whereas the Jukes–Cantor model of nucleotide substitution was employed in the ITS tree construction. Sequence alignment and phylogenetic analyses were performed using the MEGA 7.0 software package (Tamura et al. 2011; Kumar et al. 2016)

The vast majority of winter *Synechococcus* strains (14 out of 17) fall into the Bornholm Sea cluster and subalpine cluster II, and none of the winter isolates are affiliated with subcluster 5.2 (Xu et al. 2015; Fig. 7A and 6B). This result supports the earlier finding that the Chesapeake Bay contains distinct picocyanobacterial populations between summer and winter (Cai et al. 2010). One winter strain CBW1008 is in the CB7 cluster, and the other two strains appear novel. Although the phylogenetic patterns based on the 16S rRNA gene and ITS sequences do not always agree to each other, the clustering of winter strains to the major groups (i.e., Bornholm Sea cluster and Subalpine cluster II) is consistent (Figs. 7A and 6B). Interestingly, many winter Chesapeake Bay isolates are closely related to picocyanobacteria isolated from the Baltic Sea, subalpine waters, and Arctic Sea, suggesting a common origin of cold-adapted *Synechococcus*. The Bornholm Sea cluster contains a group of closely related picocyanobacteria isolated from the Baltic Sea (Ernst et al. 2003). This cluster contains six strains isolated from the Baltic Sea at two sampling sites where salinity was 7 and 9, respectively. They were closely related to *Cyanobium* spp. PCC7001 and PCC9005. Nine of our seventeen winter isolates were closely related to the members in the Bornholm Sea cluster, suggesting that similar picocyanobacterial genotypes can be present in the Chesapeake Bay and Baltic Sea. Five other winter isolates cluster in subalpine cluster II, which also contains many members isolated from the Baltic Sea. A large proportion of picocyanobacteria (39 of 46) isolated from the Baltic Sea was closely related to WH5701 in subalpine cluster II (Haverkamp et al. 2009). It appears that most of our winter picocyanobacterial isolates are closely affiliated with many picocyanobacteria isolated from the Baltic Sea, suggesting a similar niche adaptation of picocyanobacteria between these two large estuarine environments. Noticeably, none of the Baltic Sea picocyanobacterial isolates are closely related to Chesapeake Bay summer *Synechococcus* strains (i.e., CB0101 and CB0205) in subcluster 5.2. This discrepancy could be related to the different cultivation methods or latitudes. The metagenomic analysis showed that many contigs recovered from the Baltic Sea are closely related to subcluster 5.2 *Synechococcus*, especially CB0101 and CB0205 (Larsson et al. 2014). CB0101 and CB0205 are the two representative strains in subcluster 5.2. A systemic comparison between the Chesapeake Bay and the Baltic Sea can deepen our understanding of the ecological distribution of picocyanobacteria in the estuarine ecosystem.

There was a difference in cultivation methods used in earlier studies. The culture medium used to isolate the Baltic Sea *Synechococcus* contains 1 part of ASN III and 3 parts of BG11 with a salinity of 6 (for culture media see Rippka et al. 1979). We used the SN medium (Waterbury et al. 1986) to isolate Chesapeake Bay cyanobacteria, and the salinity of SN media was adjusted to reflect the salinity of water samples (Chen et al. 2004). We followed the pour plating method (Brahamsha 1996) to increase the plating efficiency. Culture isolation can be biased by the methods used. A more systematic study with the same cultivation method should be considered in future collaboration. The Baltic Sea is a brackish water system, similar to the Chesapeake Bay in many ways. The average salinity of the Baltic Sea is 6, and the low salinity is mainly caused by large freshwater input and limited exchange with the North Sea (Rodhe and Winsor 2002). However, the Baltic Sea has a higher latitude (53°N to 66°N latitude) and lower average temperature than Chesapeake Bay. In addition, the Baltic Sea has a larger water volume, deeper water column, and much longer residence time of water (25 years vs 9 months) compared to the Chesapeake Bay. Despite this, we have seen the overlap in picocyanobacterial isolates between the Chesapeake Bay and the Baltic Sea.

Isolation and characterization of Chesapeake Bay picocyanobacteria from both warm and cold seasons shed light on the taxonomy, physiology, and genetic diversity of estuarine *Synechococcus*. The Bay *Synechococcus* spp. exhibit a wider range of salt and thermal tolerance compared to their open ocean counterparts. It appears that subcluster 5.2 *Synechococcus* dominate in summer, while the Bornholm Sea cluster and subalpine cluster II picocyanobacteria prevail in winter, in the upper Chesapeake Bay. A more systematic survey is still needed to comprehensively understand picocyanobacterial biogeography across the whole Bay over time and space.

## A link between the Chesapeake Bay and freshwater picocyanobacteria

The phyletic proximity between freshwater and some marine picocyanobacteria has been seen in earlier studies based on the 16S rRNA gene phylogeny (Honda et al. 1999; Robertson et al. 2001), likely reflecting the evolutionary connection between freshwater and marine picocyanobacteria. Marine cyanobacteria evolved from cyanobacteria habituated in the terrestrial or freshwater system on the early Earth (Sanchez-Baracaldo et al. 2005; Sánchez-Baracaldo 2015). In the freshwater and brackish water, several deeply branched lineages of picocyanobacteria such as Bornholm Sea cluster, *Cyanobium* gracile cluster (Group A), Lake Biwa, subalpine cluster I (Group B), subalpine cluster II, etc., were identified, and they appeared to form a monophyletic group next to marine *Synechococcus* (Crosbie et al. 2003; Ernst et al. 2003). Later studies showed that subcluster 5.2 and these non-marine lineages also share a common ancestor (Callieri et al. 2013; Everroad and Wood 2012; Huang et al. 2014; Sánchez-Baracaldo et al. 2008; Xu et al. 2015). A broader subcluster 5.2 has been proposed to include freshwater picocyanobacteria based on the phylogenomic analysis (Cabello-Yeves et al. 2017; Di Cesare et al. 2018; Sánchez-Baracaldo et al. 2019). This large phylogenetic cluster that encompasses freshwater, brackish and marine picocyanobacteria has been called *Cyanobium* (Ernst et al. 2003), non-marine picocyanobacteria ‘*Cyanobium*’ (Everroad and Wood 2012); subcluster 5.2 (Cabello-Yeves et al. 2017, 2022; Di Cesare et al. 2018), *Cyanobium*/*Synechococcus* subcluster 5.2 (Sánchez-Baracaldo et al. 2019), *Cyanobium* (SC5.2) (Doré et al. 2020). When more freshwater and brackish water picocyanobacteria genomes were available, a broader subcluster 5.2 emerged. The original subcluster 5.2 with many coastal and estuarine *Synechococcus* was lost and replaced by larger subcluster 5.2 defined by phylogenomic analysis. This generates confusion on the definition of subcluster 5.2 (see later section).

While the close evolutionary relationship between subcluster 5.2 and many non-marine lineages is clear, a more meaningful cluster name should be used to avoid confusion with original subcluster 5.2 which mainly contains *Synechococcus* from brackish water and coastal estuaries (Ahlgren and Rocap 2012; Chen et al. 2006; Choi and Noh 2009; Huang et al. 2012, 2014; Hunter-Cevera et al. 2016; Jing et al. 2009; Xia et al. 2015; 2017; Zufia et al. 2022).

**Fig. 8:**
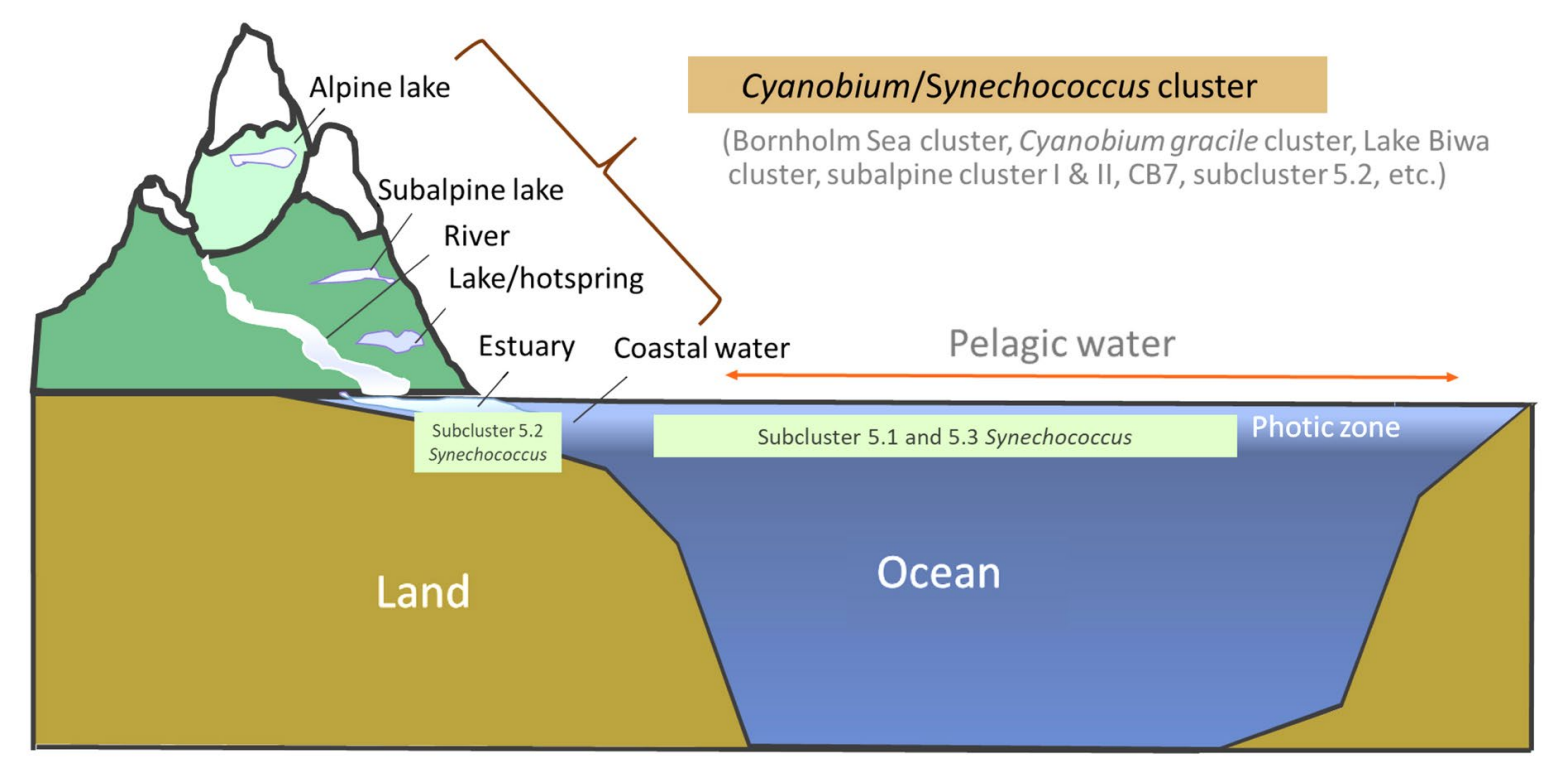
A proposal of the *Cyanobium/Synechococcus* cluster to include picocyanobacteria from diverse non-marine habitats ranging from the estuary to the alpine lake. This cluster is expected to include many deeply branched subclusters of picocyanobacteria from non-marine environments. The original subcluster 5.2 is one of three marine *Synechococcus* subclusters first established by Herdman et al. (2001) and remains unchanged in this paradigm

The author proposed to use the *Cyanobium/Synechococcus* cluster to show the monophyletic relationship between subcluster 5.2 and non-marine picocyanobacteria (Fig. 7A). This naming system is adopted from *Cyanobium*/*Synechococcus* subcluster 5.2 used by Sánchez-Baracaldo et al. (2019), but drops the term subcluster 5.2. The use of the *Cyanobium/Synechococcus* cluster is meaningful in many ways. It avoids confusion with the original subcluster 5.2 which has been established for marine and estuarine *Synechococcus*. The *Cyanobium/Synechococcus* cluster contains many deeply branched lineages that reflect specific niche adaptation to salinity, temperature, and other environmental factors. Subcluster 5.2 is one of these lineages and should be kept in its original setting because *Synechococcus* strains in this subcluster are frequently found in the estuarine and coastal regions. They are more related to marine *Synechococcus* regarding their RuBisCO gene phylogeny (Chen et al. 2004). Subcluster 5.2 represents the transitional ecotype between freshwater and marine picocyanobacterial, and is parallel to subclusters 5.1 and 5.3. The use of the *Cyanobium/Synechococcus* cluster is more meaningful for a broader cluster (not a subcluster) because: (1) members in this cluster are diverse in genomic architecture; (2) they contain different genera and species names, and (3) they can be found in rivers, lakes, hot springs, subalpine and alpine lakes, brackish water, etc. Figure 8 illustrates diverse habitats and phylogenetic lineages covered by the *Cyanobium/Synechococcus* cluster. More efforts are needed to understand the taxonomy and molecular systematics of this large cluster. Future studies should be warranted to integrate extensive ecological diversity data into the new genome-based taxonomic systems.

## 16S rRNA gene vs. ITS phylogeny

Phylogenetic analyses based on 16S rRNA or ITS sequences have been widely used to understand the genotypes and ecotypes of aquatic picocyanobacteria in the past 20 years. Novel genotypes and ecotypes of picocyanobacteria in the natural environment have been described based on these two gene markers. Phylogenetic trees of picocyanobacteria based on the 16S rRNA gene and ITS can be compared in Figs. 7A and 6B. The topology of the ITS tree is different from that of the 16S rRNA gene. Different from the 16S rRNA gene-based phylogeny, the ITS phylogeny failed to include all freshwater and estuarine clades under the *Cyanobium/Synechococcus* cluster. Subclusters 5.1 and 5.3 are more evolutionarily cognate based on both trees, but the ITS phylogeny does not support the Cyanobium/Synechococcus cluster. The discrepancy seen between ITS and 16S rRNA genetic markers is not surprising. The ITS region is highly variable regarding amplicon length and nucleotide sequence. The ITS genetic marker is more appropriate to distinguish closely related genotypes/ecotypes within a particular niche-adapted group but less suitable to explore the evolutionary relationship across distantly related organisms.

Many clusters or subclusters are deeply branched based on the ITS phylogeny, suggesting that diverse phyletic lineages are present in different habitats. Many CBW strains share high ITS sequence homology with strains isolated from the Baltic Sea in the Bornholm Sea cluster, first defined by Ernst et al. (2003). Members in the Bornholm Sea cluster may represent a group of unique *Cyanobium* spp. with large genomes and high GC content, as reflected by the genomes of CBW1002 and CBW1006 (Fucich et al. 2021a). This group of picocyanobacteria (subalpine cluster II) likely thrives in brackish water with relatively low temperatures. Subalpine cluster II is another well-defined lineage that contains many picocyanobacteria isolated from the Baltic Sea (Haverkamp et al. 2009). The marine strain WH5701 is affiliated with subalpine cluster II based on both ITS and 16S rRNA gene-based phylogenies (Chen et al. 2006; Ernst et al. 2003; Haverkamp et al. 2009; Fig. 7A and 6B), and does not belong to subcluster 5.2 *Synechococcus* as originally defined. Subalpine cluster II may contain a group of *Cyanobium* spp. with relatively large genomes and high GC content, as shown by the genome sequence of CBW1107 (Fucich et al. 2021b). Both trees support the close relationship between subcluster 5.2 and the Lake Biwa cluster. More genome sequences from the *Cyanobium*/*Synechococcus* cluster are needed to better understand the evolution and ecological adaptation of freshwater, estuarine, and marine picocyanobacteria.

## Genome sequencing of Chesapeake Bay *Synechococcus*

Genomic sequencing and comparative genomics of picocyanobacteria have significantly advanced our understanding of their ecological adaptation and molecular evolution (Dufresne et al. 2008, Palenik et al. 2003, 2006; Scanlan et al. 2009). The number of sequenced genomes of picocyanobacteria has increased dramatically in the past 20 years. Currently, 552 *Synechococcus* spp. genomes, 1182 *Prochlorococcus* spp. genomes, and 144 *Cyanobium* spp. genomes are available in GenBank (https://www.ncbi.nlm.nih.gov/). More genomes from marine picocyanobacteria have been sequenced compared to freshwater picocyanobacteria. With the support from the Gordon and Betty Moore Foundation Marine Microbiology Initiative, the draft genomes of two CB *Synechococcus* strains (CB0101 and CB0205) were sequenced in 2009, with accession numbers NZ_ADXL00000000 and NZ_ADXM00000000, respectively. CB0101 and CB0205 were chosen to represent clades CB4 and CB5 of subcluster 5.2. The draft genome sequence of CB0101 was reported in 2014 (Marsan et al. 2014), and the complete genome sequence of CB0101 was announced recently (Fucich et al. 2019). CB0101 represents the first entire genome sequence for subcluster 5.2.

*Synechococcus* strain CB0101 has been used as a model strain for Chesapeake Bay picocyanobacteria as its closely related isolates are commonly found in the Bay during the summertime. Strain CB0101 has been used to understand the ecophysiology of estuarine picocyanobacteria and to isolate cyanobacterial viruses from the Chesapeake Bay (Wang 2007; Wang and Chen 2008; Wang et al. 2011). CB0101 is more resilient than the coastal and open-ocean *Synechococcus* WH7805 in wide ranges of salinity, temperature, nutrient, and metal concentrations (Marsan 2016). The genome of CB0101 contains many genes related to the transportation, storage, use, and exportation of metals, especially copper, nickel, cobalt, and magnesium. The presence of these genes indicates its extensive capacity to sense and respond to changes in the Chesapeake Bay (Fucich et al. 2019; Marsan et al. 2014).

Recently, complete genomes of five winter CB *Synechococcus* isolates (CBW1002, CBW1004, CBW1006, CBW1107, and CBW1108) have been sequenced, and the genomic analysis has shown that these winter strains contain more genes involved in cold and other stress responses (Fucich 2020; Fucich et al. 2021a, 2021b). These five strains were chosen to represent three known phyletic lineages (Bornholm Sea cluster, Subalpine cluster II, and CB7) and a new strain. All five winter strains contain relatively high copy numbers of desaturase, chaperone, and transposase genes compared to coastal and open-ocean *Synechococcus*. These three gene categories are responsible for maintaining membrane fluidity, protein stability, and horizontal gene transfer, respectively, suggesting that they can be crucial to the niche adaptation of picocyanobacteria to the estuarine ecosystem (Fucich 2020; Fucich et al. 2021a). Genome sizes of these five winter strains range from 3.20 Mb to 3.86 Mb, with CBW1002 and CBW1006 among the largest genome sizes for picocyanobacteria (~ 3.8 Mb). The CBW1002 and CBW1006 genomes represent the first complete genome in the Bornholm Sea cluster which includes many winter CB isolates and a few Baltic Sea isolates (Figs. 7A and 6B). The large genome size and high GC content (> 65%) of CBW1002 and CBW1006 suggest that they could be more related to *Cyanobium* than *Synechococcus* (Fucich et al. 2021a). CBW1002 and CBW1006 contain 59 and 35 transposase genes, respectively, suggesting that maintaining genetic plasticity is essential to these two winter strains (Fucich et al. 2021a). The genome of CBW1107 also represents the first complete genome for Alpine subcluster II which contains many picocyanobacteria isolated from high-altitude lakes, the Baltic Sea, and winter Chesapeake Bay (Fucich et al. 2021b). In addition, winter CB strains tend to have more toxin-antitoxin genes than the coastal and open-ocean *Synechococcus*, with CBW1108 having the highest number of toxin-antitoxin genes (*n* = 80) in all known picocyanobacterial genomes (Fucich 2020).

## Toxin–antitoxin genes in picocyanobacteria

Toxin–antitoxin (TA) modules were discovered in persister cells in response to antimicrobial therapy (Lewis 2010). TA systems consist of a toxin that can arrest cell growth and a cognate antitoxin that acts as an antidote to the toxin’s activity (Unterholzner et al. 2013). TA systems are present in nearly every bacterial chromosome (Fraikin et al. 2020). TA genes are mobile cassettes and multiple copies of TA genes are present in free-living prokaryotes (Pandey and Gerdes 2005). When prokaryotic cells enter the persister state, they greatly tolerate environmental stress such as antibiotics and nutrient starvation. The self-regulating TA mechanism differs from genetic mutation, and the resulting non-dividing or persistering cells can survive harsh environments until the stress is removed (Balaban 2011; Page and Peti 2016). Four main types of TA systems (Type I to IV) have been identified based on their methods of action (Harms et al. 2018), with the Type II TA system being the most well-studied. Type II systems are characterized by a protein–protein interactive system where the antitoxin suppresses the action of the toxin through direct interaction (Pandey and Gerdes 2005).

While TA systems have been studied extensively in human-associated bacteria, little is known about the presence of TA systems in cyanobacteria. TA systems have been predicted in cyanobacteria including *Synechocystis* PCC6803 (Kaneko et al. 2003), *Microcystis aeruginosa*, and *Synechococcus* WH8102 (Makarova et al. 2009), and on the pANL plasmid in *Synechococcus* PCC7942 (Chen et al. 2016). Eleven TA gene pairs were identified in *Synechococcus* WH8102, and no TA genes were found in *Prochlorococcus marinus* MIT9313 (Makarova et al. 2009). In general, no systematic survey of TA genes in picocyanobacterial genomes was performed, and we know little about TA systems and their ecological role in marine and freshwater picocyanobacteria.

Analysis of the CB0101 genome sequence unveiled seven pairs of type II TA genes in its chromosome (Marsan et al. 2017). The transcriptomic study of CB0101 reveals a tight coupling between the upregulation of particular toxins, such as relE, with simulated stress conditions, suggesting that TA systems could be an important genetic feature for estuarine *Synechococcus* to adapt to a highly variable environment like Chesapeake Bay (Marsan et al. 2017). We recently investigated 71 complete *Synechococcus* and *Prochlorococcus* genomes to understand TA systems’ prevalence in marine and freshwater picocyanobacteria (Fucich and Chen 2020). TA systems (Type II) were predicted in 27 of 33 (81%) *Synechococcus* strains, but none of the 38 *Prochlorococcus* strains contain TA genes. *Synechococcus* strains with larger genomes tend to contain more TA systems. The number of TA pairs varies from 0 to 42 in *Synechococcus* strains isolated from various environments (Fucich and Chen 2020). Linear correlations between the genome size and the number of putative TA systems in coastal and freshwater *Synechococcus* were established, respectively (*r*^2^ = 0.9152, *p* < 0.00001 and *r*^2^ = 0.8296, *p* < 0.005). In general, open-ocean *Synechococcus* contains no or few TA systems, while coastal and freshwater *Synechococcus* contains more TA systems. Our survey shows that TA systems are widely present in many freshwater, coastal, and estuarine *Synechococcus*. Inheritance of more TA genes in freshwater and coastal *Synechococcus* could be an important mechanism for them to survive in changing environments. The open ocean is a relatively stable environment, it is interesting that all *Prochlorococcus* strains lack TA genes despite their genome sizes varying from 1.7 to 2.6 Mb. The five CB winter *Synechococcus* contain 29–80 TA genes with complex association networks (Fucich 2020). The stability of the environment and genome size both influence the presence of TA genes in picocyanobacteria.

The prevalence of TA genes in freshwater and estuarine environments and the absence of TA genes in *Prochlorococcus* and open-ocean *Synechococcus* imply an interesting environmental selection on TA systems. If marine cyanobacteria evolved from freshwater cyanobacteria, it is plausible that TA genes were lost when marine *Synechococcus* occupied the ocean. *Synechococcus* adapted to the estuarine environment could serve as excellent models to understand the evolution of TA systems in unicellular cyanobacteria. Although abundant TA genes exist in non-marine picocyanobacteria, the biological and ecological function of TA systems in cyanobacteria remains largely unexplored. There are other stress response systems in cyanobacteria. We still do not know how TA systems coordinate with other stress response systems.

## Conclusion and prospects

This review summarizes what we have learned from estuarine *Synechococcus*, mainly based on the Chesapeake Bay studies in the past 20 years. In the summer, the main Chesapeake Bay contains mostly estuarine and marine type *Synechococcus*. In the Bay, subcluster 5.2 *Synechococcus* thrive in warm months/summer, but freshwater *Synechococcus* and *Cyanobium* become dominant in cold months/winter. In general, picocyanobacteria isolated from the Bay are more environmentally tolerant than coastal and open-ocean *Synechococcus* spp. It is believed that strong environmental gradients in the Chesapeake Bay select for estuarine picocyanobacteria with diverse pigment types, genotypes, and ecotypes. Genome sequences of these Chesapeake Bay picocyanobacteria have shed light on the ecological adaptation of estuarine picocyanobacteria. The role of toxin–antitoxin genes in picocyanobacteria deserves a close look in the future. The five complete genome sequences of winter CB picocyanobacteria reveal new evidence of stress response capabilities for estuarine picocyanobacteria, while comparative genomics and phylogenomic analysis with CBW genomes are currently underway.

The number of sequenced genomes of picocyanobacteria has increased greatly in the past 20 years. Phylogenomics has been used to redefine the taxonomic position of *Synechococcus* at the order, family, genus, and species levels, changing the traditional paradigm of picocyanobacterial classification (Coutinho et al. 2016a; Sánchez-Baracaldo et al. 2019; Salazar et al. 2020; Komárek et al. 2020; Tschoeke et al. 2020). A new genus Parasynechococcus that includes marine *Synechococcus* strains has been proposed based on the genome-based phylogeny (Coutinho et al. 2016a). The link between the genomes of cultured cyanobacteria and environmental genomes such as Tara Oceans’ metagenomes could provide insight into the biogeography of picocyanobacteria (Coutinho et al. 2016b; Walter et al. 2017). The rapidly increasing cyanobacterial genome sequences together with metagenomes will greatly deepen our understanding of the taxonomy, evolution, and ecology of cyanobacteria.

The new naming system should consider and credit earlier work (Komárek et al. 2020). The discrepancy still exists in phylogenomic clustering and nomenclature, especially for phylotypes or genotypes defined in earlier ecological studies. It is clear that subcluster 5.2, brackish and freshwater picocyanobacteria share a common ancestor, and can be grouped based on the 16S rRNA gene or genome-based phylogeny. The author suggests that the *Cyanobium/Synechococcus* cluster rather than subcluster 5.2 is a more appropriate cluster name to embrace the related members of *Cyanobium* and *Synechococcus* that have been discovered in freshwater, estuarine, and marine ecosystems (Fig. 8). Doing so will keep subcluster 5.2 as a small lineage that contains many isolates and genotypes of *Synechococcus* found in coastal estuaries, similar to the lineages like Bornholm Sea cluster, Subalpine cluster I and II, Lake Biwa, etc., which were defined in the earlier studies. These lineages help separate picocyanobacterial microdiversity related to specific aquatic environments.

While taxonomic classification and molecular evolution of picocyanobacteria based on the genomic information is timely, efforts are still needed to better interpret the ecological diversity of picocyanobacteria. Large datasets based on marker genes (i.e., 16S rRNA gene, ITS, *rpoC*, etc.) have been accumulated in the past 20 years and provided a deep understanding of the biogeographic distribution of picocyanobacteria in the coastal estuary and pelagic ocean, primarily based on the structure of marine subclusters 5.1, 5.2, and 5.3. Approaches based on gene markers are suitable for analyzing many ecological samples at a much lower cost than metagenomics. To better integrate gene-based and genome-based phylogeny, further studies are needed to include all the well-defined genotypes and ecotypes of picocyanobacteria.

Freshwater and estuarine habitats are complex, dynamic, and subject to human activities. A systematic study based on molecular tools is needed to explore the picocyanobacterial community across aquatic ecosystems over time and space. Isolation of cyanobacteria from unexplored habitats will continue to uncover new phylotypes. The combination of culture and molecular methods will provide a comprehensive insight into the phylogenetic diversity and evolution of freshwater, estuarine, and marine picocyanobacteria.

## Data Availability

Data will be made available on request.
